# A novel AI-based score for assessing the prognostic value of intra-epithelial lymphocytes in oral epithelial dysplasia

**DOI:** 10.1038/s41416-024-02916-z

**Published:** 2024-11-30

**Authors:** Adam J. Shephard, Hanya Mahmood, Shan E. Ahmed Raza, Syed Ali Khurram, Nasir M. Rajpoot

**Affiliations:** 1 Tissue Image Analytics Centre, Department of Computer Science, University of Warwick, Coventry, UK; 2 School of Clinical Dentistry, University of Sheffield, Sheffield, UK

**Keywords:** Oral cancer detection, Oral cancer detection, Computer science, Prognostic markers

## Abstract

**Background:**

Oral epithelial dysplasia (OED) poses a significant clinical challenge due to its potential for malignant transformation and the lack of reliable prognostic markers. Current OED grading systems do not reliably predict transformation and suffer from considerable observer variability. Recent studies have highlighted that peri-epithelial lymphocytes may play an important role in OED malignant transformation, with indication that intra-epithelial lymphocytes (IELs) may also be important.

**Methods:**

We propose a novel artificial intelligence (AI) based IEL score from Haematoxylin and Eosin (H&E) stained Whole Slide Images (WSIs) of OED tissue slides. We determine the prognostic value of our IEL score on a digital dataset of 219 OED WSIs (acquired using three different scanners), compared to pathologist-led clinical grading.

**Results:**

Our IEL scores demonstrated significant prognostic value (C-index = 0.67, *p* < 0.001) and were shown to improve both the binary/WHO grading systems in multivariate analyses (*p* < 0.001). Nuclear analyses confirmed the positive association between higher IEL scores, more severe OED and malignant transformation (*p* < 0.05).

**Conclusions:**

This underscores the potential importance of IELs, and by extension our IEL score, as prognostic indicators in OED. Further validation through prospective multi-centric studies is warranted to confirm the clinical utility of IELs.

## Background

Head and neck cancer encompasses a diverse group of malignancies originating from the upper aerodigestive tract, including the oral cavity, nasal cavity, pharynx, larynx, salivary glands and sinuses [1]. Among these, oral squamous cell carcinoma (OSCC) stands as one of the most prevalent subtypes, predominantly affecting the oral mucosa and accounting for a significant proportion of head and neck cancer cases [1]. OSCC arises from the squamous epithelial cells lining the oral cavity and is strongly associated with risk factors such as tobacco use and alcohol consumption [2]. Characterised by aggressive local invasion and potential for regional and distant metastasis, OSCC poses considerable challenges in diagnosis and management. Combination therapy approaches including surgery, radiation therapy, and chemotherapy are often employed [3], which even if successful, are associated with functional problems including masticatory, speech and swallowing impairments, drastically affecting quality of life [4]. Prognosis for advanced stage OSCC is poor, having a five-year survival rate of just 40% [5]. This drastically increases with early diagnosis to 80–90% [5], highlighting the huge benefits of early detection.

Oral cancer lesions are typically preceded by a group of lesions termed oral potentially malignant disorders (OPMDs) including homogeneous/non-homogeneous leucoplakia (white/white-red speckled lesions) or erythroplakia (red lesions) [2, 6]. Biopsies of the lesions enable microscopic examination by histopathologists to determine the presence or absence of oral epithelial dysplasia (OED) or cancer. Lesions of the oral mucosa exhibiting dysplasia are statistically more likely to transition into OSCC than non-dysplastic lesions [7].

The histopathological grading of Haematoxylin and Eosin (H&E) stained tissue using the World Health Organisation (WHO, 2017 [8]) classification system remains the current accepted practice for diagnosis and risk stratification of OED lesions. This system categorises OED into three grades: mild, moderate, and severe, based on the presence, severity, and location of various cytological and architectural histological features (28 in total [9, 10]). However, this approach has been widely criticised for its significant intra- and inter-observer variability and its limited predictive ability for malignant transformation risk, which can impact patient management. An alternate binary grading system has been proposed to improve the reproducibility of grading [11, 12]. This system classifies lesions as either low- or high-risk based on the number of cytological and architectural features outlined in the WHO criteria. Mahmood et al. [13] showed the utility of various of these pathologist-assigned histological features in prognostic models. However, overall, studies have shown that both the three-tier and binary grading systems suffer from significant variability and unreliability [14, 15]. This underscores the need for more objective and reproducible methods and *features* (markers) for grading OED that can better predict the risk of malignant transformation.

Recent advancements in digital pathology have facilitated the digitisation of histology slides into whole slide images (WSIs) through high-resolution digital scanners. This has spurred significant growth in computational pathology [16, 17]. Concurrently, the evolution of new deep learning techniques has complemented both pathology and radiology, enabling the automation of pipelines and demonstrating the potential of deep learning in predicting patient outcomes [16–18]. In the emerging area of computational pathology, deep learning has been applied to automatically segment epithelium across various histology images (e.g. oral, cervical, prostate) [19–23] and to further segment and classify individual nuclei within WSIs [24, 25]. In the context of OED, our previous work has used deep learning to segment dysplasia [20] and also the oral epithelium into sub-regions: the lower basal layer, the middle epithelial layer, and the superior keratin layer [19, 21]. These methods have even been used to predict OED malignant transformation, based on either deep [26] or nuclear features [19, 27]. Thus, deep learning tools offer a potential avenue for reducing grading variability while ensuring consistency across sites in informing treatment decisions [28, 29].

Computational pathology has not only allowed researchers to replicate and automate pathology workflows, but also to aid in biomarker discovery. Bashir et al. [26] developed a pipeline to predict malignant transformation in OED. They discovered a positive association between increased peri-epithelial lymphocytes (PELs) and malignant transformation. Further, Shephard et al.’s [19] work also suggested the potential association between both PELs and intra-epithelial lymphocytes (IELs) and malignant transformation in OED. These studies highlight the need for further exploration and validation of the role of IELs in OED.

IELs are small, round mononuclear white blood cell lymphocytes found within the epithelial layer in the paracellular space between epithelial cells [30]. They are found in the skin and within the epithelial layer lining the intestine, lungs, reproductive tract and oral cavity, and are typically thought to be T lymphocytes [30, 31]. In the gastrointestinal (GI) tract, they are components of gut-associated lymphoid tissue. Within normal mice, there is one IEL per 5–10 epithelial cells in the small intestine. In human duodenal biopsies, healthy individuals usually have less than 5–10 IELs per 100 epithelial cells; however, this number can increase significantly, and is a hallmark of coeliac disease [31]. In the oral cavity, the inflammatory response and the role of IELs is poorly understood [32]. As yet, neither PELs nor IELs are used as markers within the WHO or binary grading system.

While the role of lymphocytes in cancer immunity is well-documented, their significance in dysplasia remains underexplored. Therefore, we present an in-depth exploration of IELs within OED, and investigate the prognostic value of a digital IEL score. This approach aligns with other automated methods within the computational pathology community, such as Tumour-Infiltrating Lymphocyte (TIL) scoring [33] and Mitosis Counting (MC) [34]. We aim to elucidate the prognostic utility of AI-generated IEL scores in OED, utilising advanced image analysis techniques to quantify lymphocytic infiltration within the epithelium. Through comprehensive evaluation of the correlation between IEL scores and clinical outcomes using a relatively large dataset (in the context of OED), we explore the potential of the proposed IEL score as a prognostic indicator. Given results in previous work [19], we hypothesise that IEL count is related to malignant transformation in OED. In the spirit of reproducibility, we also release the full inference pipeline for generating our AI-generated IEL scores, based on H&E-stained WSIs from oral tissue sections, adamshephard/oed_iel_scoring (github.com).

## Methods

### Study data

The study data consisted of a retrospective cohort of histology tissue sections (dating 2008 to 2016 with minimum five-year follow-up data) collected from the Oral and Maxillofacial Pathology archive at the School of Clinical Dentistry, University of Sheffield, UK. After initial microscopic inspection of the tissue sections by a Consultant Pathologist (SAK), newly cut 4 µm sections of the selected cases were obtained from formalin fixed paraffin embedded blocks and stained with H&E for analysis.

A purposive sampling method was employed, selecting consecutive cases from the pathology archive within the specified time period. Inclusion criteria required sufficient epithelial tissue, high-quality staining, and complete follow-up data. Cases were excluded if they had ulceration or overlying candidal infection, or involved HPV-related OED or verrucous lesions; based on morphology on H&E. Cases with clinical oral lichen planus (OLP) or coincidental OLP were also excluded from this analysis. Further, cases with insufficient tissue, poor staining quality, or incomplete follow-up data were also excluded. Care was taken to ensure a reasonable mix of grades were included. The sample size was determined by the availability of eligible cases within the specified time period.

To ensure diagnostic consistency, multiple certified consultant pathologists (PMS, PMF, KH, DJB), initially evaluated the cases using the WHO grading system. The cases were then blindly re-evaluated by an Oral & Maxillofacial Pathologist (SAK) and an Oral Surgeon specialising in OED analysis (HM) to confirm the WHO (2017) grade and to assign binary grades. Any diagnostic disagreements were resolved through team discussion.

A total of 219 slides from 188 patients, were collected and digitised into high-resolution WSIs at 40× objective power. This was done using one of three scanners: NanoZoomer S360 (Hamamatsu Photonics, Japan; 0.2258 mpp), Aperio CS2 (Leica Biosystems, Germany; 0.2520 mpp), Pannoramic 1000 (P1000, 3DHISTECH Ltd, Hungary; 0.2426 mpp). Clinical data, including patient age (at time of diagnosis), sex, intraoral site, OED grade (using binary and WHO 2017 systems) and transformation status, were extracted from patient records and hospital clinical systems. Transformation was defined as the progression of a dysplastic lesion to OSCC at the same clinical site within the follow-up period, with time to transformation measured in months. Among the cohort, 42 patients (with 49 WSIs) experienced malignant transformation. An overview of the dataset is provided in Table 1.

**Table 1:** Overview of OED samples included in this study.

Characteristic	
OED Cases, *n*	188
OED Slides, *n*	219
Median Age^a^ (IQR)	63 (53–73)
Sex, *n* (%)	
Female	95 (43)
Male	124 (57)
Site, *n* (%)	
Buccal Mucosa	29 (13)
Tongue	97 (44)
Floor of Mouth	41 (19)
Other	52 (24)
WHO grade, *n* (%)	
Mild	79 (36)
Moderate	77 (35)
Severe	63 (29)
Binary grade, *n* (%)	
Low-risk	134 (61)
High-risk	85 (39)
Transformation, *n* (%)	49 (22)
Median Transformation-free Survival Months (IQR)	78 (60–108)
Scanner, *n* (%)	
Aperio CS2	41 (19)
NanoZoomer S360	98 (45)
P1000	80 (37)

All provided statistics are at the slide-level.

^a^Median age at OED diagnosis.

The study received ethical approval (18/WM/0335), and all methods were conducted in accordance with the Declaration of Helsinki. We adhered to the REMARK [35] guidelines and have included the checklist as Supplementary Information.

### Deep learning framework

#### Dysplasia and nuclear segmentation

Since dysplastic changes may not be widespread across the entire tissue section in a slide, the first step of the AI pipeline involved identification and localisation of the dysplastic tissue regions for semantic segmentation. To achieve this, we used a pretrained Transformer [20] (based on Trans-UNet [36]) that automatically detects and segments the different dysplastic regions in a H&E-stained oral tissue WSIs. Further, an additional pretrained CNN-based HoVer-Net+ model [19, 21] was used to segment the epithelium and the individual nuclei across each WSI. This model classifies nuclei within the epithelium as being “epithelial” or “other” nuclei. In OED tissue, aside from epithelial nuclei, we primarily expect to see immune cells, most of which are likely to be lymphocytes. Thus, for the purpose of this study we have classified these “other” nuclei as IELs. To visually confirm this assertion, we have segmented nuclei in 15 regions of interest (ROIs) by both the HoVer-Net+ model and Cerberus [37, 38]. Cerberus is a multi-task CNN trained to segment and classify nuclei, amongst other tasks, within colorectal WSIs. It was chosen as it is the only other model we are aware of that can segment and classify nuclei as being either lymphocytes, neutrophils or eosinophils. We then counted the number of lymphocytes vs neutrophils and eosinophils within the epithelium by Cerberus vs “other” nuclei by HoVer-Net + , and asked an expert pathologist (SAK) to further comment on these results. We further provide Pearson correlations between epithelial and lymphocyte cell counts.

#### Intra-epithelial lymphocyte (IEL) scoring

For IEL scoring, we counted the number of IELs within the dysplasia regions alone, and we used these counts to generate the following IEL scores:

1. The IEL Count (IEL-C)—the **number of IELs per 100 dysplastic epithelial cells**, within the **entire** dysplastic region of the WSI
2. The IEL Peak Count (IEL-PC)—the **maximum number of IELs per 100 dysplastic epithelial cells**, in **any given area** of dysplasia (here, chosen to be a patch of size 512×512, at 1.0 mpp resolution)

**Fig. 1:**
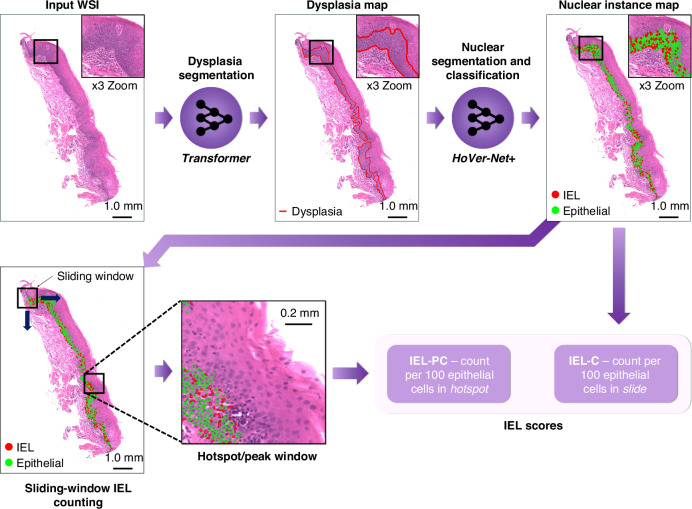
Overview of the pipeline used to generate IEL scores. An input WSI first goes through the Trans-UNet model for dysplasia segmentation. Following this, we perform nuclear segmentation using HoVer-Net+ . We then generate the IEL-C scores based on the IELs and epithelial nuclei detected in the dysplastic regions. For the IEL-PC score we find the window with the highest value for that score, using a sliding window approach.

In Fig. 1, we provide an overview of the proposed analytical pipeline used to generate our IEL scores. We further provide two additional IEL scores – the IEL Index (IEL-I) and IEL Peak Index (IEL-PI)—based on the number of IELs per unit area of dysplasia, in the Supplementary Material (pp 2).

### Clinicopathologic analysis

We first performed statistical analyses to compare various clinicopathological parameters and our AI-based IEL scores between cases that exhibited transformation and those that did not. We used chi-square tests for sex, site, scanner and binary grade (with odds ratio OR as effect size). For age, WHO grade and IEL scores we used a Mann–Whitney U test with point-biserial correlation *r*_*pb*_, as effect size.

Next, we aimed to additionally see how our IEL scores correlated with the clinicopathological parameters aside from transformation. Thus, for sex and binary grade we used a Mann–Whitney U test (point-biserial correlation *r*_*pb*_ effect size). For lesion site and scanner vendor we used Kruskal-Wallis H tests (eta-squared η^2^ effect size), followed by post-hoc Mann–Whitney U tests if significant (point-biserial correlation *r*_*pb*_ effect size). For age and WHO grade, Spearman’s corelation *ρ* was used, with *p*-values calculated via permutation tests. We then performed post-hoc Mann–Whitney U test between different combinations of WHO grades.

### Survival analysis

Survival analyses were conducted to assess the prognostic significance of the IEL scores in predicting transformation-free survival. Cases were split into low- and high-risk groups based on whether their IEL score was lesser/greater than the mean IEL score. We used the mean IEL score, owing to the imbalance in the number of cases transforming to malignancy (22%), and therefore did not necessarily require a 50–50 split in low-/high-risk groups. Kaplan-Meier curves were generated, and log-rank tests were used to determine the statistical significance of this stratification (for IEL scores, WHO and binary grades). We used concordance index (C-Index) to measure the rank correlation between the scores and patients’ survival time.

To further test the effect of the proposed IEL scores on current clinical grading systems, we provide two further scores, the binary-IEL+ and binary-IEL- scores. For binary-IEL+ , we upgrade any low-risk cases to high-risk based on whether the IEL score is high (above the mean value), whereas for binary-IEL-, we downgrade any high-risk cases to low-risk based on whether the IEL score is low (below the mean value). We provide further WHO-IEL- and WHO-IEL+ scores in the Supplementary Material (pp 2), where cases are upgraded/downgraded by a single grade, based on high/low IEL scores. A univariate Cox proportional hazards (PH) model was employed to determine the prognostic utility of the IEL scores compared to the WHO grade, binary grade, sex, age and lesion site, to predict transformation-free survival. Transformations were right censored at eight years, as most events occurred within this timeframe (median survival time of 6.5 years). We therefore, additionally used the hazard ratio (HR) and *p*-value generated from the univariate analyses as further metrics for evaluation. For reporting, we focus on the *p*-value from the PH model analyses, over that of the log-rank test, since both tests share the same assumptions, but Cox PH models allow for the use of continuous exposure variables [39]. However, for completeness we also provide the log-rank *p*-value with the Kaplan-Meier curves. Following this, we also conducted multivariate analyses to test the effect of combining the various clinical and digital parameters on transformation-free survival.

### Comparison to other dysplastic features and prognostic models

For a subset of 109 cases, three expert pathologists additionally assessed each slide to determine whether 12 different OED histological (both architectural and cytological) features were present (binary variable—present vs not present). This data was originally presented as part of our previous work [13], and these features include: basal cell hyperplasia, bulbous/drop shaped rete pegs, dyskeratosis, hyperchromatism, irregular surface keratin, loss of epithelial cohesion, loss of stratification, suprabasal mitoses, nuclear pleomorphism, abrupt orthokeratosis, lymphocytic band, verrucous surface. Within this subset we aimed to further test whether the IEL scores were correlated with any specific dysplastic features. A Mann–Whitney U test was therefore conducted to assess the relationship between the continuous IEL scores and the 12 histological features, and we report point-biserial correlation coefficient *r*_*bp*_ as the effect size.

Finally, we build upon our previous work [13], by reproducing the 2- and 6-point prognostic multivariate Cox PH models. The 2-point prognostic model included the following features: bulbous/drop shaped rete pegs, and loss of epithelial cohesion. Whilst the 6-point model included: bulbous/drop shaped rete pegs, hyperchromatism, loss of epithelial cohesion, loss of stratification, suprabasal mitoses, nuclear pleomorphism. These specific six features were chosen in-line with the original study, where they were associated with a greater incidence of transformation. We modify both of these models to additionally incorporate the IEL score, WHO/binary grade, age, sex and site, to see the impact of the additional features. We compare the performance of these models based on the C-Index alone.

## Results

### Nuclear classification performance

**Fig. 2:**
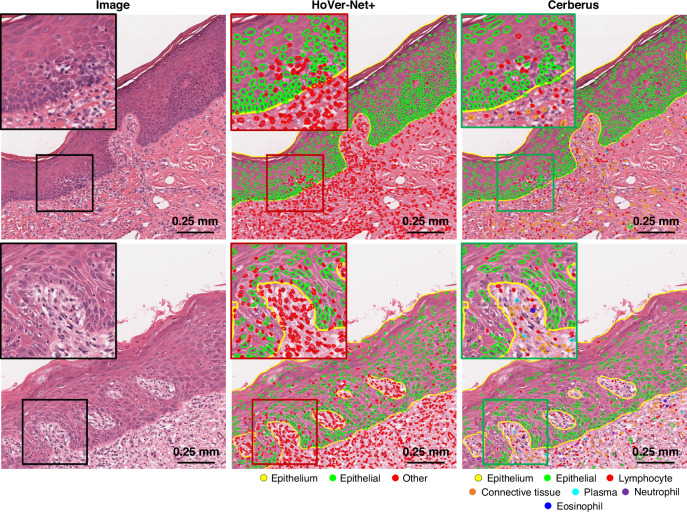
Visualisation of HoVer-Net+ vs Cerberus nuclear segmentation and classification performance. Note, the epithelium segmentation (yellow) on both HoVer-Net+ /Cerberus images is output by the HoVer-Net+ model.

To confirm the assertion that the majority of “other” nuclei within the epithelium are IELs, within 15 ROIs we compared the counts of lymphocytes to neutrophils and eosinophils, as counted by Cerberus. Qualitatively, an expert pathologist (SAK), agreed that the “other” nuclei within the epithelium as detected by HoVer-Net+ (and Cerberus) were mostly lymphocytes. From a quantitative standpoint, across the epithelium of these 15 ROIs, Cerberus counted 947 lymphocytes, 15 neutrophils and 5 eosinophils. Thus, the predicted count of lymphocytes within the epithelium (by Cerberus), outnumbers those of both neutrophils and eosinophils by a factor of ~50. See Fig. 2 for a visual comparison of HoVer-Net+ and Ceberus’ nuclear segmentation and classification results. Here, we display two sample ROIs, where HoVer-Net+ ’s segmentation appear to be more sensitive. For detailed metrics regarding nuclear segmentation/classification performance of both Cerberus and HoVer-Net+ , we direct the reader to their respective papers [19, 37]. Pearson correlation showed a high correlation between epithelial cell counts (*r* = 0.98 *p* < 0.001) and intra-epithelial lymphocyte cell counts (*r* = 0.72 *p* = 0.002) by both Cerberus and HoVer-Net+ .

### Clinicopathologic analysis

We first tested the clinicopathological associations between cases that did and did not transform to malignancy. We found no significant association between cases that transformed and age (non-transformed: median M = 63 (interquartile range IQR = 52–72); transformed: M = 63 (55–74); *r*_*pb*_ = 0.00, *p* = 0.94), sex (non-transformed: nr. males = 97; transformed: males = 27; OR = 0.92, *p* = 0.81), lesion site (non-transformed: nr. buccal mucosa = 24, tongue = 70, front of mouth FOM = 37, “other” = 39; transformed: buccal mucosa = 5, tongue = 27, FOM = 4, “other” = 13; *p* = 0.11) or scanner vendor (non-transformed: nr. Aperio CS2 = 33, NanoZoomer S360 = 79, P1000 = 58; transformed: Aperio CS2 = 8, NanoZoomer S360 = 19, P1000 = 22; *p* = 0.39). With regards to lesion site, the buccal mucosa had an OR of 0.54 compared to the tongue, 1.91 compared to the floor of mouth (FOM), and 0.62 compared to “other” areas. The tongue then had OR of 3.54 compared to the FOM, and 1.17 compared to “other” areas, whilst the FOM had an OR of 0.33 compared to “other” areas. With regards to scanner vendor, the OR for transformation with Aperio CS2 cases compared to NanoZoomer S360 cases was 1.01, and compared to the P1000 was 0.64. The OR for P1000 cases compared to NanoZoomer S360 was 1.57. Higher grade cases were significantly associated with transformation for both binary (OR = 10.38, *p* < 0.001) and WHO grades (*r*_*pb*_ = 0.29, *p* < 0.001).

**Fig. 3:**
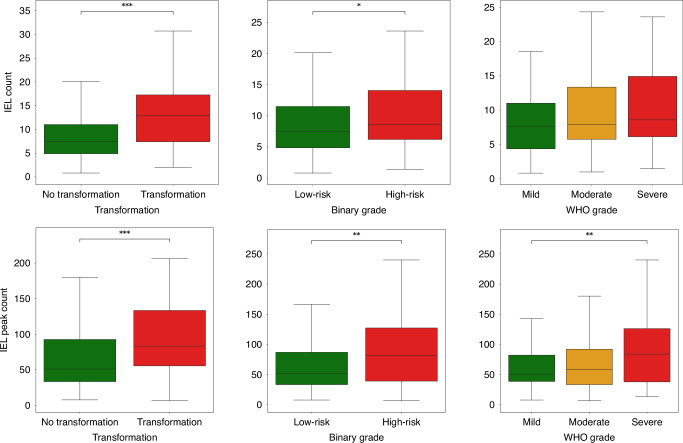
The distribution of IEL scores across OED cases based on transformation and grade. Boxplots showing the distribution of IEL scores in OED cases according to: transformation status (left), where transforming cases are red and not transforming are green; binary grade (middle), where low-risk cases are green and high-risk are red; and WHO grade (right), where mild cases are green, moderate orange, and severe are red. The top row is for the IEL-C score and the bottom row the IEL-PC score.

Both IEL scores (IEL-C and IEL-PC) were found to not be normally distributed (*p* < 0.001) via a Shapiro-Wilk test, and thus non-parametric statistical tests were employed throughout this section. First, we compared the various IEL scores in cases that transformed to malignancy against those that did not (see Fig. 3). We found significantly higher IEL scores for cases that transformed, with large effect sizes. We found the largest difference between groups in the IEL-C (low-risk: M = 7.39 (4.87–11.02); high-risk: M = 12.95 (7.42 – 17.28); *r*_*pb*_ = 0.28, *p* < 0.001) and IEL-PC scores (low-risk: M = 51.00 (33.33–92.64); high-risk: M = 83.33 (55.42–133.33); *r*_*pb*_ = 0.30, *p* < 0.001).

We additionally showed how the scores varied by grade (see Fig. 3). Our IEL scores correlate well with the binary grade, showing high-risk cases to have generally higher IEL-C (low-risk: M = 7.52 (4.86–11.50); high-risk: M = 8.58 (6.20–14.07); *r*_*pb*_ = 0.09, *p* = 0.04) and IEL-PC (low-risk: M = 52.32 (33.33–86.85); high-risk: M = 81.82 (39.13–127.27); *r*_*pb*_ = 0.26, *p* = 0.003) scores. However, the effect sizes are only small-to-moderate in size, suggesting that the IEL scores are still adding new prognostic information. For the WHO grade, we see that higher grades were not significantly associated with higher IEL-C (*ρ* = 0.13, *p* = 0.05), but were significantly associated with higher IEL-PC (*ρ* = 0.18, *p* = 0.008) scores. Post-hoc analyses found severe OED cases to have significantly higher IEL-PC scores than mild cases (mild: M = 50.00 (38.52–82.22); severe: M = 84.00 (37.80–126.14); *r*_*pb*_ = 0.28, *p* = 0.008).

We additionally compared our IEL-C and IEL-PC scores to other clinical variables including age, sex, lesion site and scanner. We found no significant association between age and IEL-C (*ρ* = 0.02, *p* = 0.82) or IEL-PC (*ρ* = 0.04, *p* = 0.56). No significant correlations were found between sex and IEL-C (female: M = 8.39 (5.48–13.51); male: M = 7.90 (5.25–12.26); *r*_*pb*_ = 0.02, *p* = 0.70) or IEL-PC (female: M = 60.00 (33.83–104.06); male: M = 56.20 (37.34–104.89); *r*_*pb*_ = 0.06, *p* = 0.48) scores. Similarly, no significant correlations were found between lesion sites and the IEL-C (η^2^ = 0.00, *p* = 0.35) and IEL-PC scores (η^2^ = 0.01, *p* = 0.97). With regards to scanner vendor, no significant difference was found between scanner vendor and IEL-C (η^2^ = 0.02, *p* = 0.06) or IEL-PC (η^2^ = 0.02, *p* = 0.07) scores.

### Survival analysis

**Fig. 4:**
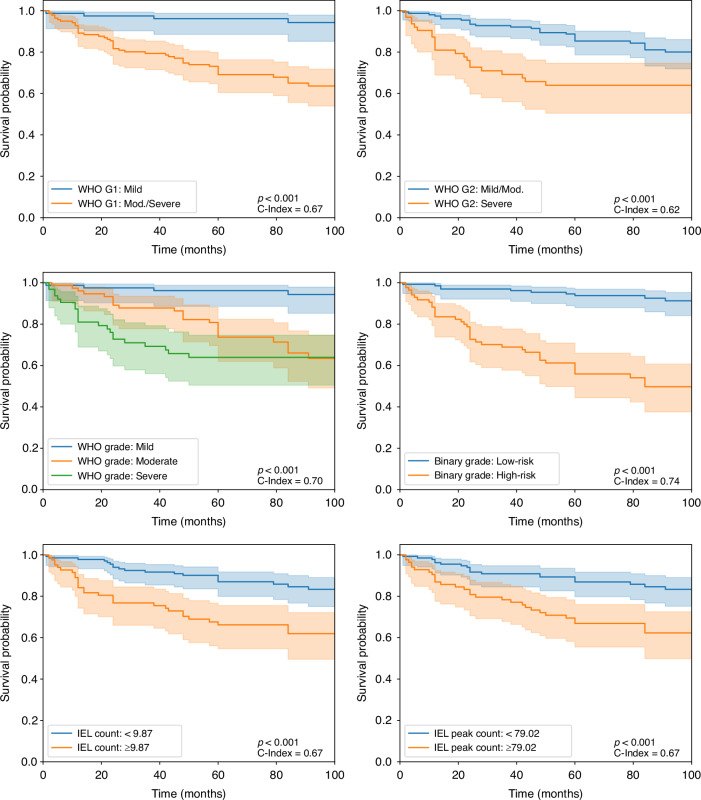
Kaplan-Meier survival curves for pathologist grades and IEL scores. Survival curves for WHO and binary grade (top and middle rows) and  IEL-C/IEL-PC scores (bottom row).

We compared the IEL scores to the other grading systems through Kaplan-Meier curves (see Fig. 4) to demonstrate their prognostic utility. The binary grading scheme showed the clearest separation between low- and high-risk cases (C-index = 0.74, *p* < 0.001). For ease of comparison, we divided the WHO grades into two groupings. For WHO G1, where mild cases were compared against moderate and severe cases combined; we observed a clear separation between cases (C-index = 0.67, *p* < 0.001). For WHO G2, where mild and moderate cases combined were compared against severe cases, we observed a slightly less clear stratification (C-index = 0.62, *p* < 0.001). For the IEL scores, cases were split into low- and high-risk groups according to the mean IEL score. Both the IEL-C and IEL-PC scores gained a C-index of 0.67 (both *p* < 0.001).

**Fig. 5:**
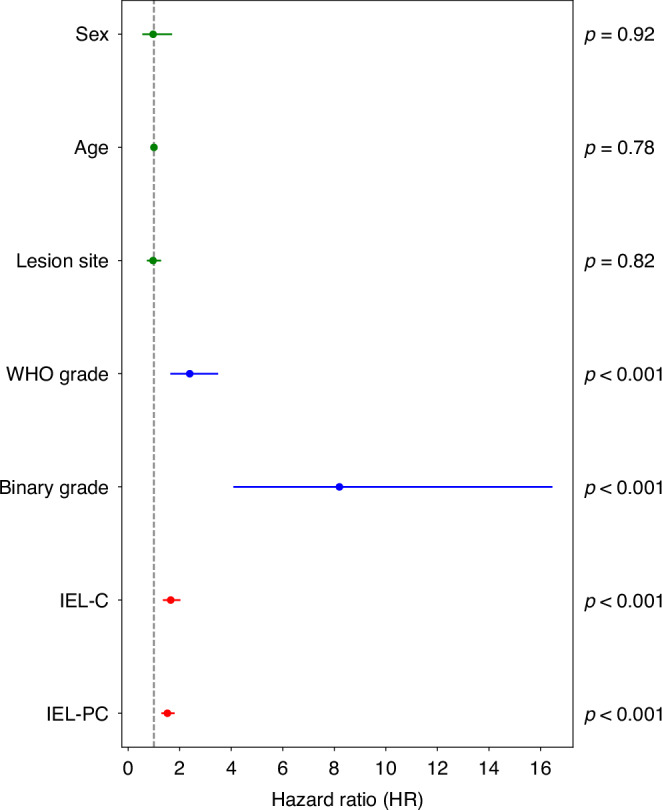
Forest plot of hazard ratios (HRs) for the clinical parameters (green), grades (blue), and proposed IEL scores (IEL-C and IEL-PC, red), from univariate cox proportional hazards (PH) models. 95% lower and upper confidence intervals also given, along with *p*-values from the PH models.

We present the results from the univariate Cox PH models for clinical and digital parameters/scores in Table 2. This table illustrates that factors such as sex, age, and lesion site appear to not significantly effect survival. We see that IEL-binary+ and IEL-binary- grades give slightly increased C-Indexes when compared to the IEL scores alone, but are reduced when compared to the binary grade. Thus, we do not include these scores in any further analyses. We have illustrated the HRs and their 95% confidence intervals (CIs) for the remaining variables in a forest plot (see Fig. 5). One can see high HRs for the binary grade and WHO grades, with slightly lower HRs for the IEL scores.

**Table 2:** Univariate analysis of clinical and digital parameters.

Parameter	HR [95% CI]	*p*	C-Index
Sex	0.97 [0.55–1.70]	0.916	0.50
Age	1.00 [0.98–1.02]	0.783	0.51
Site	0.97 [0.73–1.28]	0.819	0.53
WHO Grade	2.39 [1.63–3.49]	**< 0.001**	0.70
WHO G1	8.06 [2.90–22.44]	**< 0.001**	0.67
WHO G2	2.56 [1.46–4.50]	**0.001**	0.62
Binary Grade	8.20 [4.08–16.46]	**< 0.001**	0.74
IEL Scores			
IEL-C	1.65 [1.35–2.03]	**< 0.001**	0.67
IEL-PC	1.52 [1.29–1.80]	**< 0.001**	0.67
IEL-Binary+			
IEL-C	12.14 [3.77–39.04]	**< 0.001**	0.69
IEL-PC	10.27 [3.69–28.59]	**< 0.001**	0.70
IEL-Binary-			
IEL-C	7.44 [4.19–13.20]	**< 0.001**	0.69
IEL-PC	5.13 [2.91–9.05]	**<0.001**	0.67

Reported metrics are from a univariate Cox proportional hazards model. HR is the hazard ratio, where the 95% confidence interval (CI) is given in brackets. WHO G1 is mild vs moderate/severe cases. WHO G2 is mild/moderate vs severe cases.

We additionally show the multivariate analyses in Table 3, comparing the effect of combining the above clinical and digital parameters on transformation-free survival, in terms of C-Index. The prediction performance increased when adding clinical variables (i.e. age, sex, site) to both the WHO and binary grades by between 1 and 2%. Similarly, the addition of the IEL-C and IEL-PC scores, separately, increased the model performance by between 4 and 6%, with the highest C-Index = 0.82 for combining the binary grade, age, sex, lesion site and IEL-C score. Thus, these analyses demonstrate the prognostic utility of the IEL-C and IEL-PC scores, and the potential utility of adding IEL information to the grading system.

**Table 3:** Multivariate analysis of clinical and digital parameters, compared in terms of C-Index.

	Model (C-Index)	Model + IEL-C score (C-Index)	Model + IEL-PC score (C-Index)
WHO	0.703	0.758	0.756
WHO + Age + Sex + Site	0.716	0.764	0.754
Binary	0.740	0.812	0.804
Binary + Age + Sex + Site	0.758	**0.820**	0.803

**Fig. 6:**
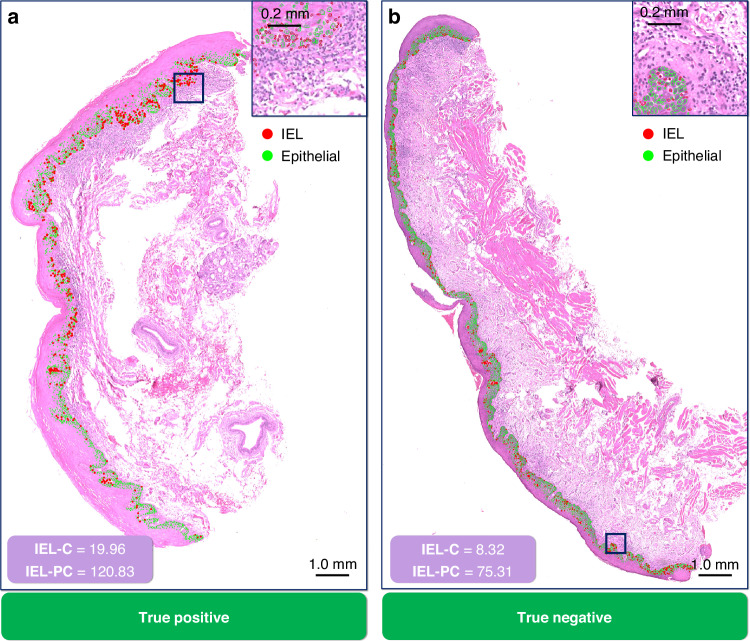
Visualisation of segmented nuclei within the dysplastic epithelium. Each panel shows a different WSI with the nuclear detections overlaid, where green dots are epithelial nuclei, and red dots are IELs. We additionally display the IEL-C and IEL-PC score for each slide, and the hotspot used to generate the IEL-PC score. Panel (**a**) shows a WSI where the scores were high, and it transformed (i.e. a true positive); whilst (**b**) shows a WSI where the scores were low, and it did not transform (i.e. a true negative). Within these analyses the cutoff values for the IEL-C and IEL-PC scores were 9.87 and 79.02, respectively.

Finally, we visualise some of the results of this study, comparing cases that did transform with a high IEL-C (and IEL-PC) value, respectively, to a case that did not transform with a low IEL-C (and IEL-PC; see Fig. 6). These images also show the hotspots used to generate the IEL-PC scores. Overall, we see the hotspots tend to focus on the basal layer of the epithelium, which tend to have the highest density of IELs. Visibly, the case with higher IEL-C/IEL-PC appears to have many more IELs than the case with a low score.

### Comparison to other dysplastic features and prognostic models

For the subset of 109 cases where we additionally had manually assigned histological features from the work of Mahmood et al. [13], we first tested the correlation of both the IEL-C and IEL-PC score with each of the 12 histological features. Note, for the 12 histological features rated by three pathologists, consensus agreement was determined where 2 out of 3 pathologists had determined the feature to be either present or not.

We first looked for correlation between IEL scores and the various dysplastic features. We found the strongest correlation between lymphocytic bands and both the IEL-C score (present: M = 13.42 (9.14–18.87); not present: M = 7.34 (4.98–9.13); *r*_*pb*_ = 0.47, *p* < 0.001) and the IEL-PC score (present: M = 89.64 (59.13–141.84); not present: M = 47.37 (33.33–73.77); *r*_*pb*_ = 0.30, *p* = 0.002). Medium-strong correlations were also found between loss of epithelial cohesion and both the IEL-C score (present: M = 9.21 (7.56–17.24); not present: M = 8.09 (5.19–11.50); *r*_*pb*_ = 0.21, *p* = 0.031) and the IEL-PC score (present: M = 73.77 (47.22–143.71); not present: M = 54.07 (33.33–88.12); *r*_*pb*_ = 0.32, *p* < 0.001). Weak-moderate, but significant correlations were found between the IEL scores and hyperchromatism, irregular surface keratin, loss of stratification, suprabasal mitoses, and abrupt orthokeratosis. No significant correlations were found between the IEL scores and basal cell hyperplasia, bulbous rete pegs, nuclear pleomorphism, dyskeratosis and verrucous surface. Additional details for all comparisons are summarised in Table 4.

**Table 4:** Summary of point-biserial correlations between pathologist-assigned histology features and digital IEL scores.

Histological feature	IEL score	Feature present M (IQR)	Feature not present M (IQR)	*r*_*pb*_	*p*
Basal cell hyperplasia	IEL-C	8.23 (5.41–12.72)	8.92 (7.27–16.60)	0.14	0.14
	IEL-PC	54.69 (34.67–105.51)	59.09 (40.35–88.37)	0.09	0.36
Bulbous/drop shaped rete pegs	IEL-C	8.57 (6.03–13.05)	8.05 (6.01–13.24)	0.14	0.15
	IEL-PC	54.45 (37.73–103.24)	58.82 (35.48–92.00)	0.06	0.50
Dyskeratosis	IEL-C	8.58 (5.59–15.07)	8.29 (6.26–11.87)	0.07	0.45
	IEL-PC	61.90 (45.45–120.83)	54.42 (34.09–90.91)	0.12	0.23
Hyperchromatism	IEL-C	9.44 (6.87–16.47)	7.68 (5.05–10.28)	0.19	**0.045**
	IEL-PC	59.40 (43.72–117.40)	54.35 (33.03–79.08)	0.19	0.05
Irregular surface keratin	IEL-C	8.05 (5.96–11.70)	8.91 (6.03–15.95)	0.20	**0.04**
	IEL-PC	53.33 (33.71–95.45)	59.41 (44.70–106.00)	0.10	0.31
Loss of epithelial cohesion	IEL-C	9.21 (7.56–17.24)	8.09 (5.19–11.50)	0.21	**0.03**
	IEL-PC	73.77 (47.22–143.71)	54.07 (33.33–88.12)	0.32	**< 0.001**
Loss of stratification	IEL-C	9.05 (6.18–15.40)	7.92 (5.40–10.99)	0.10	0.28
	IEL-PC	80.91 (45.20–141.84)	52.63 (33.33–70.00)	0.27	**0.004**
Suprabasal mitoses	IEL-C	8.90 (7.27–14.01)	7.91 (5.08–11.35)	0.02	0.87
	IEL-PC	60.00 (38.71–130.68)	54.45 (33.59–84.35)	0.23	**0.01**
Nuclear pleomorphism	IEL-C	8.27 (6.13–12.49)	8.73 (5.69–13.33)	0.02	0.87
	IEL-PC	53.33 (33.26–122.92)	57.98 (39.69–91.73)	0.05	0.61
Abrupt orthokeratosis	IEL-C	8.20 (5.47–13.11)	8.75 (6.09–13.94)	0.17	0.07
	IEL-PC	53.59 (33.33–89.00)	58.82 (4.44–114.29)	0.21	**0.03**
Lymphocytic band	IEL-C	13.42 (9.14–18.87)	7.34 (4.98–9.13)	0.47	**< 0.001**
	IEL-PC	89.64 (59.13–141.84)	47.37 (33.33–73.77)	0.30	**0.002**
Verrucous surface	IEL-C	8.20 (6.38–11.52)	8.58 (5.40–14.27)	0.09	0.33
	IEL-PC	54.61 (33.33–97.73)	55.42 (38.92–102.63)	0.07	0.45

M is the median IEL score, IQR is the interquartile range of the IEL score. r_pb_ is the point-biserial correlation coefficient, and the p-value is from a Mann–Whitney U test.

Next, we tested the performance of both 2- and 6-point prognostic models (in terms of C-Index) following the addition of various clinical parameters and the IEL-C and IEL-PC scores (see Table 5). For both the 2- and 6-point models the C-Index increased with the addition of clinical parameters (age, sex, site) and the binary grade. However, the addition of the WHO grade yielded mixed effects. Adding both the IEL-C and IEL-PC scores yielded an increase in C-Index in the prognostic models across all combinations. The addition of both the binary grade and the IEL-C score improved performance the most from the baseline in both the 2-point and 6-point model (C-Index = 0.75 and C-Index = 0.80, respectively) to C-Index = 0.88 and C-Index = 0.88, respectively.

**Table 5:** Comparison of the prognostic performance for the 6- and 2-point models, combined with various clinical variables, with and without the inclusion of the digital IEL-C and IEL-PC scores.

	Model (C-Index)	Model + IEL-C score (C-Index)	Model + IEL-PC score (C-Index)
6-point	0.797	0.858	0.808
6-point + Age + Sex + Site	0.810	0.858	0.820
6-point + WHO grade	0.796	0.850	0.807
**6-point + Binary grade**	0.817	**0.878**	0.834
2-point	0.745	0.840	0.793
2-point + Age + Sex + Site	0.773	0.850	0.805
2-point + WHO grade	0.765	0.853	0.791
**2-point + Binary grade**	0.795	**0.880**	0.827

## Discussion

In this study, we investigated the prognostic potential of IELs derived from digital pathology in OED, a precursor lesion to OSCC. We highlighted the challenges associated with the current diagnostic and prognostic methods for OED, emphasising the need for more objective and reproducible approaches to predict the risk of malignant transformation. We addressed this gap by employing advanced deep learning techniques to automate the segmentation of dysplastic regions and IELs within oral tissue WSIs, and introduced two IEL scores, the IEL-C and IEL-PC, as potential prognostic indicators. Finally, we tested the prognostic utility of these scores in various survival models.

Our findings demonstrated the promising prognostic utility of the proposed digital IEL scores in predicting transformation-free survival in OED patients. Notably, the IEL scores generated based on the number of IELs per dysplastic epithelial cells (i.e. IEL-C and IEL-PC scores), when compared to the IEL-I and IEL-PI (see Supplementary Material Table S1) showed the strongest association with clinical outcomes, as evidenced by their higher concordance indices and significant separation of low- and high-risk cases. The success of these count-based scores is perhaps unsurprising as they mimic the IEL scores used previously in the literature, typically seen in duodenal biopsies for studying coeliac disease [40, 41]. We additionally suggest that the IEL-C was more prognostic when compared to the IEL-PC (with a higher hazard ratio, and C-Indices in multivariate analyses), as any spurious IEL detections by the deep learning models, could result in a substantially higher (or indeed lower) IEL score in any given patch, resulting in an incorrect hotspot region for calculating the IEL-PC. By contrast, incorrect detections would be diluted in the IEL-C.

Comparison with traditional grading systems revealed that the binary grading scheme demonstrated clearer stratification of risk groups compared to the WHO grading system. Both of the proposed IEL scores showed some stratification of high- and low-risk groups, but with slightly less clear separation, and lower hazard ratios than the binary and WHO grades. Furthermore, statistical analyses and boxplots illustrated the significance of IEL scores in distinguishing between cases that progressed to malignancy and those that did not, with higher IEL scores consistently observed in transformed cases.

Our analysis showed that upgrading or downgrading a case’s binary grade (see Supplementary Materials Table S1 for WHO grade) based on high or low IEL scores (e.g. binary-IEL+ and binary-IEL-) did not improve the prognostic value compared to the original binary grading system. This outcome is not entirely surprising, as this rudimentary approach reduces the continuous IEL score to a binary variable, losing much of the detailed information it contains. However, when incorporated into a multivariate model with other clinical parameters, the inclusion of IEL scores provided additional prognostic information beyond both grading systems. This suggests that relying on a simple binary cut-off in IEL score to adjust grades may not be sufficient. Instead, IEL scores should be interpreted in a more nuanced manner by clinicians, integrating their granularity into more sophisticated grading systems, or indeed models, to fully capture their prognostic value.

Our additional analyses correlating the digital IEL scores to various pathologist-defined histological features in a subset of our dataset (*n* = 109), revealed further associations between IEL scores and histological features. Specifically, the IEL scores had the highest correlation with a subepithelial lymphocytic band being present on the H&E slide. This finding supports the notion that IEL density increases with a more general increased immune response. Unsurprisingly, no significant correlation was found between IEL scores and other histological features such as basal cell hyperplasia, bulbous rete pegs, nuclear pleomorphism, dyskeratosis and verrucous surface. Within this smaller cohort we further modified our previously described 2- and 6-point prognostic models [13] and found that by incorporating the IEL-C score, both the 2- and 6-point model C-Index improved (by between 5 and 10%). Even when the binary/WHO grading systems were included, large improvements were seen with the inclusion of the IEL score.

The potential positive association between inflammatory response and malignant transformation in OED is an interesting finding, challenging the conventional notion of immune cell infiltration being a favourable prognostic factor, which is often seen in cancer. However, we suggest that our finding may not be counterintuitive, with a higher immune response in dysplasia being indicative that a mechanism may already be underway (unseen on the simple H&E slide) in the action of transforming the OED lesion into cancer. Furthermore, these results are consistent with previous studies that have observed a higher abundance of immune cells, including both IELs and PELs, in more dysplastic OED cases. Gannot et al. [42] noted increased immune cell infiltration in tongue lesions progressing to OSCC. Similarly, both Fitzpatrick et al. [43] and Hidalgo et al. [32] found a substantial number of OED cases to have band-like inflammatory cell infiltrate underlying the epithelium (i.e. PELs) and infiltrating the epithelium (i.e. IELs). Recent evidence has suggested a higher density of PELs in cases undergoing malignant transformation [26]. This was further supported by Shephard et al. [19], who additionally found a potential association between both PELs and IELs and malignant transformation. We would, however, like to additionally highlight that not all immune cells in the peri-epithelium, as found in these studies [19, 26], will be lymphocytes, and there may also be other cell types such as plasma cells, histiocytes, and eosinophils etc. present.

The role of these immune cells in OED lesions is still subject to debate, with studies suggesting that severe dysplasia often become progressively infiltrated with immune suppressive myeloid cells and regulatory T cells (Treg), with Treg infiltration associated with an increased risk of malignant progression [44, 45]. Yet, despite this, OED lesions are often strongly infiltrated with CD8 + T lymphocytes, that may act to reverse this immunosuppressive microenvironment [45, 46]. Generally, CD8 + T-cells are understood to be crucial in tumour immunity, while CD4 + T-cells are known to enhance tumour invasion [47]. A recent review [48], highlighted the switch from CD8+ to CD4+ to be associated with malignant progression, and concluded that a more thorough understanding of the role of the immune system is required to ultimately translate understanding to precision oncology.

Our findings demonstrate that AI-based IEL scores are prognostic, supporting our initial hypothesis. Moreover, integrating these digital IEL scores into existing grading systems could enhance their predictive accuracy and improve risk stratification in OED. As a future direction, we recommend further investigation into the utility of a pathologist-derived IEL score to validate its clinical utility. Our study highlights the potential of computational pathology and deep learning in identifying novel prognostic biomarkers and refining diagnostic and therapeutic strategies in head and neck cancer. Additionally, future work should focus on constructing a nomogram that visualises the contribution of IELs to enhance OED grading and prognostication, enabling more tailored patient management.

The authors recognise some limitations regarding this study. Despite the work being based of a sizeable OED cohort (indeed one of the largest known digital OED cohorts to date), the dataset used was still relatively small. Moreover, all data was collected retrospectively from a single tertiary centre, and thus may be subject to certain biases. This includes a potential bias towards higher-risk lesions, as tertiary referral patterns often lead to the selection of more severe cases. While we employed purposive sampling to ensure a reasonable mix of grades, there remains a natural predisposition towards higher-risk cases, as reflected in the 22% transformation rate observed in the study. It could be argued that this may reduce the generalisability of our findings to the broader OED population; however, patients are often referred and diagnosed at a late stage of the disease process.

We would have additionally liked to incorporate both lesion characteristics (e.g. size, colour, texture) and social risk factors (e.g. tobacco and alcohol consumption) in the multivariate analyses, however, we were unable to acquire this data due to the inconsistent nature of record keeping in a retrospective cohort. Further prospective validation in larger, multicentric cohorts is warranted to confirm the generalisability and robustness of our findings, and to address the issues surrounding collecting additional clinical information.

In this work, we used a pre-trained HoVer-Net+ model to segment and classify epithelial vs “other” nuclei. We have made the assertion that these “other” nuclei within the epithelium are lymphocytes, or IELs. From a biological standpoint, it is likely that most of these cells are lymphocytes, as opposed to neutrophils. Neutrophils have a distinct morphological multilobed appearance and are usually seen in the superficial keratin layer in conditions such as candidosis, erythema migrans and ulceration and not in dysplasia. Within this work, we ensured exclusion of any cases with ulceration or overlying candidal infection. Therefore, the location and the morphological appearance of the “other” nuclei within the epithelium gives us the confidence that the vast majority (if not all) of the analysed cells are lymphocytes. This was further supported by our analyses where Cerberus determined there to be 50X more lymphocytes when compared to neutrophils and eosinophils within the epithelium across 15 ROIs. Future work should train a specific deep learning model to detect IELs.

Finally, the mechanistic underpinnings of the observed associations between IEL infiltration and malignant transformation in OED warrant further investigation. Future work should focus on using immunohistochemical staining for specific subtypes of lymphocytes, such as for CD4+ and CD8 + T-lymphocytes, to better understand their role in malignant transformation. Identifying these lymphocyte subtypes will provide deeper insights into their roles in the immune microenvironment and towards malignant progression, and may help with their utility as therapeutic targets. In addition to this, it would be interesting and useful to explore the pathogenesis of OED and whether or not IEL infiltration precedes architectural disturbances, and act as an early predictor of malignancy.

In conclusion, our study contributes to the growing body of evidence supporting the role of immune cell infiltration as a potential prognostic indicator in OED. By elucidating the molecular mechanisms underlying the interactions between immune cells and dysplastic epithelial cells, we may uncover new avenues for personalised diagnostic and therapeutic strategies in head and neck oncology.

## Supplementary information

Supplemental Material

## Data Availability

Source code is made publicly available, subject to intellectual property constraints (https://github.com/adamshephard/oed_iel_scoring).

## Data availability

Data sharing is not possible due to restrictions in the ethics applications.
