# Supplementary material for: A novel AI-based score for assessing the prognostic value of intra-epithelial lymphocytes in oral epithelial dysplasia
Source: Br J Cancer. 2024 Nov 30;132(2):168–79. doi: 10.1038/s41416-024-02916-z (PMC11747091; doi:10.1038/s41416-024-02916-z)
Supplement: Supplementary file 1 — Supplemental Material [file 41416_2024_2916_MOESM1_ESM.docx]

**Supplementary Materials**

Title: A Novel AI-based Score for Assessing the Prognostic Value of Intra-Epithelial Lymphocytes in Oral Epithelial Dysplasia

Authors: Adam J Shephard, Hanya Mahmood, Shan E Ahmed Raza, Syed Ali Khurram, Nasir M Rajpoot

**Table of Contents**

[**Supplementary Methods** 2](#_Toc181090138)

[**Supplementary Results** 2](#_Toc181090139)

[***Clinicopathologic Analysis*** 2](#_Toc181090140)

[***Survival Analysis*** 3](#_Toc181090141)

[**Supplementary Discussion** 4](#_Toc181090142)

[**Supplementary Tables** 6](#_Toc181090143)

[**Supplementary Figures** 8](#_Toc181090144)

[**Supplementary References** 10](#_Toc181090145)

**Supplementary Methods**

We additionally tried two further methods for generating IEL scores:

1. The IEL Index (IEL-I) – the **number of IELs** per unit area of dysplasia, within the **entire** dysplastic region of the WSI
2. The IEL Peak Index (IEL-PI) – the **maximum number of IELs** per unit area of dysplasia in **any given area** of dysplasia (here, chosen to be a patch of size 512 x 512, at 1.0 mpp resolution)

In **Figure S1,** we provide an overview of the proposed analytical pipeline used to generate all four IEL scores. We perform the same clinicopathological and univariate survival analyses for these two IEL scores as was done for IEL-C and IEL-PC in the main manuscript.

To further test the effect of the proposed IEL scores on current clinical grading systems, we provide two further scores, the binary-IEL+ and binary-IEL- scores. For binary-IEL+, we upgrade any low-risk cases to high-risk based on whether the IEL score is high (above the mean value), whereas for binary-IEL-, we downgrade any high-risk cases to low-risk based on whether the IEL score is low (below the mean value). We provide further WHO-IEL- and WHO-IEL+ scores, where cases are upgraded/downgraded by a single grade, based on high/low IEL scores. For example, for WHO-IEL+ a mild case with a high IEL score would be upgraded to moderate, whilst a moderate case would be upgraded to severe.

**Supplementary Results**

***Clinicopathologic Analysis***

Both IEL scores (IEL-I and IEL-PI) were shown to not be normally distributed (*p* < 0.001) according to a Shapiro-Wilk test, therefore non-parametric statistical tests were used in all subsequent analyses. Initially, we compared IEL scores between cases that progressed to malignancy against those that did not (see **Figure S2**). We observed significantly higher IEL scores in cases that transformed, with moderate effect sizes (IEL-I: no transformation: Median M = 0.00039 (Interquartile range IQR = 0.000023 – 0.00062); transformation: M = 0.00056 (0.00033 – 0.00074); *r_pb_* = 0.17, *p* = 0.005; and IEL-PI: no transformation: M = 0.0023 (0.0014 – 0.0033); transformation: M = 0.0029 (0.0021 – 0.0041); *r_pb_* = 0.14, *p* = 0.007.

Further analysis examined score distributions by histological grade (see **Figure S2**), revealing that high-risk (binary grade) cases generally had increased IEL-I (low-risk: M = 0.00042 (0.00027 – 0.00063); high-risk: M = 0.00045 (0.00027 – 0.00068); *r_pb_* = 0.02, *p* = 0.047) and IEL-PI (low-risk: M = 0.0023 (0.0013 – 0.0033); high-risk: M = 0.0028 (0.0019 – 0.0037); *r_pb_* = 0.16, *p* = 0.03) scores. However, the effect sizes are only small in size, suggesting that the IEL scores are adding new prognostic information. For the WHO grade, we see that higher grades were not significantly associated with higher IEL-I (*ρ* = 0.01, *p* = 0.85) or IEL-PI (*ρ* = 0.11, *p* = 0.09) scores.

We compared our IEL-I and IEL-PI scores to other clinical variables including age, sex, lesion site and scanner. We found no significant association between age and IEL-I (*ρ* = 0.02, *p* = 0.78) or IEL-PI (*ρ* = 0.01, *p* = 0.92). Similarly, no significant correlations were found between sex and IEL-I (female: M = 0.00042 (0.00026 – 0.00067); male: M = 0.00045 (0.00029 – 0.00064); *r_pb_* = 0.03, *p* = 0.66) or IEL-PI (female: M = 0.0025 (0.0015 – 0.0034); male: M = 0.0024 (0.0016 – 0.0035); *r_pb_* = 0.04, *p* = 0.80) scores. However, significant correlations were found between lesion sites and the IEL-I (η^2^ = 0.05, *p* = 0.002) and IEL-PI scores (η^2^ = 0.03, *p* = 0.01). Post-hoc analyses found significantly higher IEL-I scores in the buccal mucosa when compared to “other” areas of the mouth (buccal mucosa: M = 0.00053 (0.00039 – 0.00063); other: M = 0.00039 (0.00018 – 0.00062); *r_pb_* = 0.17, *p* = 0.04), in the floor of mouth when compared to the tongue (tongue: M = 0.00033 (0.00018 – 0.00064); floor of mouth: M = 0.00053 (0.00039 – 0.00063); *r_pb_* = 0.25, *p* < 0.001), and in “other” areas when compared to the tongue (tongue: M = 0.00033 (0.00018 – 0.00064); other: M = 0.00047 (0.00030 – 0.00075); *r_pb_* = 0.19, *p* = 0.01). Post-hoc analyses additionally found significantly higher IEL-PI scores in “other” areas of the mouth when compared to the buccal mucosa (buccal mucosa: M = 0.0020 (0.0011 – 0.0027); other: M = 0.0028 (0.0019 – 0.0043); *r_pb_* = 0.29, *p* = 0.006), in the floor of the mouth when compared to the buccal mucosa (buccal mucosa: M = 0.0020 (0.0011 – 0.0027); floor of mouth: M = 0.0031 (0.0021 – 0.0037); *r_pb_* = 0.27, *p* = 0.008), and in “other” areas when compared to the tongue (tongue: M = 0.0024 (0.0014 – 0.0033); other: M = 0.0028 (0.0019 – 0.0043); *r_pb_* = 0.16, *p* = 0.049).

With regards to scanner vendor, significant difference were found between scanner vendor and IEL-I (η^2^ = 0.07, *p* < 0.001) and IEL-PI (η^2^ = 0.09, *p* < 0.001) scores. Post-hoc analyses found significantly higher IEL-I scores in NanoZoomer S360 cases when compared to Aperio CS2 cases (Aperio CS2: M = 0.00030 (0.00016 – 0.00056); NanoZoomer S360: M = 0.00053 (0.00033 – 0.00073); *r_pb_* = 0.30, *p* < 0.001), and when compared to P1000 cases (NanoZoomer S360: M = 0.00053 (0.00033 – 0.00073); P1000: M = 0.00039 (0.00022 – 0.00058); *r_pb_* = 0.26, *p* = 0.002). Post-hoc analyses additionally found significantly higher IEL-PI scores in NanoZoomer S360 cases when compared to Aperio CS2 cases (NanoZoomer S360: M = 0.0029 (0.0021 – 0.0040); Aperio CS2: M = 0.0020 (0.0009 – 0.0028); *r_pb_* = 0.26, *p* < 0.001), and when compared to P1000 cases (NanoZoomer S360: M = 0.0029 (0.0021 – 0.0040); P1000: M = 0.0021 (0.0013 – 0.0031); *r_pb_* = 0.30, *p* < 0.001).

***Survival Analysis***

We provide Kaplan-Meier curves for the IEL-I and IEL-PI in **Figure S3**, to demonstrate their prognostic utility, where the IEL-I score gained a C-Index of 0.63 (*p* = 0.003), and the IEL-PI score gained a C-Index of 0.61 (*p* = 0.02).

See **Table S1** for the results from the univariate Cox proportional hazard models based on the digital IEL scores. The HRs for the IEL scores are lower than that of the individual grades while still being significant, with IEL-C having the highest HR = 1.65 and showing significance (*p* < 0.001). We see that IEL-binary+ and IEL-binary- grades give slightly increased C-Indexes when compared to the IEL scores alone, but are reduced when compared to the binary grade. We see similar results for all four IEL scores with the WHO grades. However, we would like to point out that the WHO-IEL-PI+ grade gives a slightly increased C-Index of 0.71 when compared to the WHO grade (C-Index = 0.70) or the IEL-PI score (C-Index = 0.61) alone. This suggests that it is providing additional prognostic information to the WHO grade.

We additionally show the multivariate analyses **Table S2**, comparing the effect of combining the above clinical and digital parameters on transformation-free survival, in terms of C-Index. The prediction performance generally increased when adding clinical variables (i.e. age, sex, site) to both the WHO and binary grades by between 1% and 2%. Similarly, the addition of the IEL-I and IEL-PI scores, separately, increased the model performance by between 2% and 6%, with the highest C-Index = 0.81 for combining the binary grade, age, sex, lesion site and IEL-I score. Thus, these analyses demonstrate the prognostic utility of the IEL-I and IEL-PI scores, and the potential utility of adding IEL information to the grading system.

**Supplementary Discussion**

In this study, we further investigated the prognostic utility of digital intraepithelial lymphocyte (IEL) scores in predicting malignant progression in oral epithelial dysplasia (OED). Our findings indicate that the index-based IEL scores, such as IEL-I and IEL-PI, provide less prognostic value than the count-based scores, IEL-C and IEL-PC. This aligns with previous studies using IEL scores in duodenal biopsies for conditions like coeliac disease, where count-based metrics have been shown to be informative (1,2). We attribute the enhanced prognostic potential of IEL scores based on the entire slide (IEL-C and IEL-I) to their robustness against isolated detection inaccuracies by deep learning models. When IEL scores are derived from peak or hotspot regions (e.g. IEL-PI and IEL-PC), a single incorrect detection may disproportionately influence the score, leading to misleading high or low values within a patch. In contrast, slide-wide scores are less impacted by such localised errors, as erroneous detections are diluted across the broader tissue area.

Our analyses revealed significant differences in our index-based IEL scores based on lesion sites and scanner types. Post-hoc analyses showed notable variation in IEL scores between buccal mucosa, floor of mouth, and tongue lesions, suggesting that IEL density might vary according to tissue location, potentially due to lesion site-specific immune responses. Significant score differences were also observed between scanner vendors, with IEL-I and IEL-PI scores being higher on NanoZoomer S360 compared to Aperio CS2 and P1000 systems. This indicates a possible impact of scanning hardware on IEL quantification, underscoring the importance of standardised scanning protocols to ensure consistency in digital pathology.

Regarding the prognostic enhancement of clinical grading, the WHO-IEL-PI+ score provided a slight increase in predictive accuracy (C-index) over the WHO grade alone, suggesting some additive value in specific grading contexts. However, this effect was not observed with the binary grading system, indicating that the integration of IEL information may be more beneficial in certain grading frameworks than others. Our multivariate analysis demonstrated how incorporating IEL-I and IEL-PI scores as continuous variables, along with clinical factors such as age, sex, and lesion site, significantly improved the predictive performance of both the binary and WHO grading systems. The binary grade alone yielded a C-Index of 0.74, which increased to 0.80 with the addition of IEL-I, thus underscoring the value of a multivariate framework that utilises the continuous IEL scores. This finding supports the need for more sophisticated methods that combine IEL data with histological grades to enhance prognostication.

In summary, our findings support the potential utility of IEL-I and IEL-PI scores in prognostication within OED. Although their prognostic contributions are more modest compared to IEL counts scores, they offer additional insights, particularly when used alongside clinical variables. Further studies should explore optimising IEL scoring methods to enhance prognostic accuracy and assess their applicability across broader lesion sites and diverse clinical contexts.

**Supplementary Tables**

**Table S1.** Univariate analysis of additional IEL scores.

| Parameter | HR [95% CI] | *p* | C-Index |
| --- | --- | --- | --- |
| IEL Scores |  |  |  |
| IEL-I | 1.35 [1.06 – 1.72] | **0.013** | 0.63 |
| IEL-C | 1.65 [1.35 – 2.03] | **< 0.001** | 0.67 |
| IEL-PI | 1.26 [0.99 – 1.61] | 0.057 | 0.61 |
| IEL-PC | 1.52 [1.29 – 1.80] | **< 0.001** | 0.67 |
| IEL-Binary+ |  |  |  |
| IEL-I | 9.93 [3.09 – 31.95] | **< 0.001** | 0.67 |
| IEL-C | 12.13 [3.77 – 39.05] | **< 0.001** | 0.69 |
| IEL-PI | 16.48 [4.00 – 67.87] | **< 0.001** | 0.68 |
| IEL-PC | 10.27 [3.69 – 28.59] | **< 0.001** | 0.69 |
| IEL-Binary- |  |  |  |
| IEL-I | 6.71 [3.80 – 11.84] | **< 0.001** | 0.69 |
| IEL-C | 7.44 [4.19 – 13.20] | **< 0.001** | 0.69 |
| IEL-PI | 4.04 [2.23 – 7.14] | **< 0.001** | 0.64 |
| IEL-PC | 5.13 [2.91 – 9.05] | **< 0.001** | 0.67 |
| IEL-WHO+ |  |  |  |
| IEL-I | 2.95 [1.80 – 4.81] | **< 0.001** | 0.70 |
| IEL-C | 2.94 [1.84 – 4.71] | **< 0.001** | 0.70 |
| IEL-PI | 3.44 [2.04 – 5.79] | **< 0.001** | 0.71 |
| IEL-PC | 2.82 [1.79 – 4.43] | **< 0.001** | 0.70 |
| IEL-WHO+ |  |  |  |
| IEL-I | 2.77 [1.92 – 3.99] | **< 0.001** | 0.70 |
| IEL-C | 2.89 [2.00 – 4.18] | **< 0.001** | 0.70 |
| IEL-PI | 2.25 [1.59 – 3.18] | **< 0.001** | 0.70 |
| IEL-PC | 2.40 [1.70 – 3.39 | **< 0.001** | 0.69 |

*Note. Reported metrics are from a univariate Cox proportional hazards model. HR is the hazard ratio, where the 95% confidence interval (CI) is given in brackets.*

**Table S2.** Multivariate analysis of clinical and digital parameters, compared in terms of C-Index.

|  | Model  (C-Index) | Model + IEL-I score  (C-Index) | Model + IEL-PI score  (C-Index) |
| --- | --- | --- | --- |
| WHO | 0.703 | 0.742 | 0.731 |
| WHO + Age + Sex + Site | 0.716 | 0.741 | 0.731 |
| Binary | 0.740 | 0.803 | 0.780 |
| Binary + Age + Sex + Site | 0.758 | **0.805** | 0.777 |

**Supplementary Figures**





**Figure S1.** Overview of the pipeline used to generate IEL scores. An input WSI first goes through the Trans-UNet model for dysplasia segmentation. Following this, we perform nuclear segmentation using HoVer-Net+. We then generate the IEL-I and IEL-C scores based on the IELs and epithelial nuclei detected in the dysplastic regions. For the IEL-PI and IEL-PC scores we find the window with the highest value for that score, using a sliding window approach.


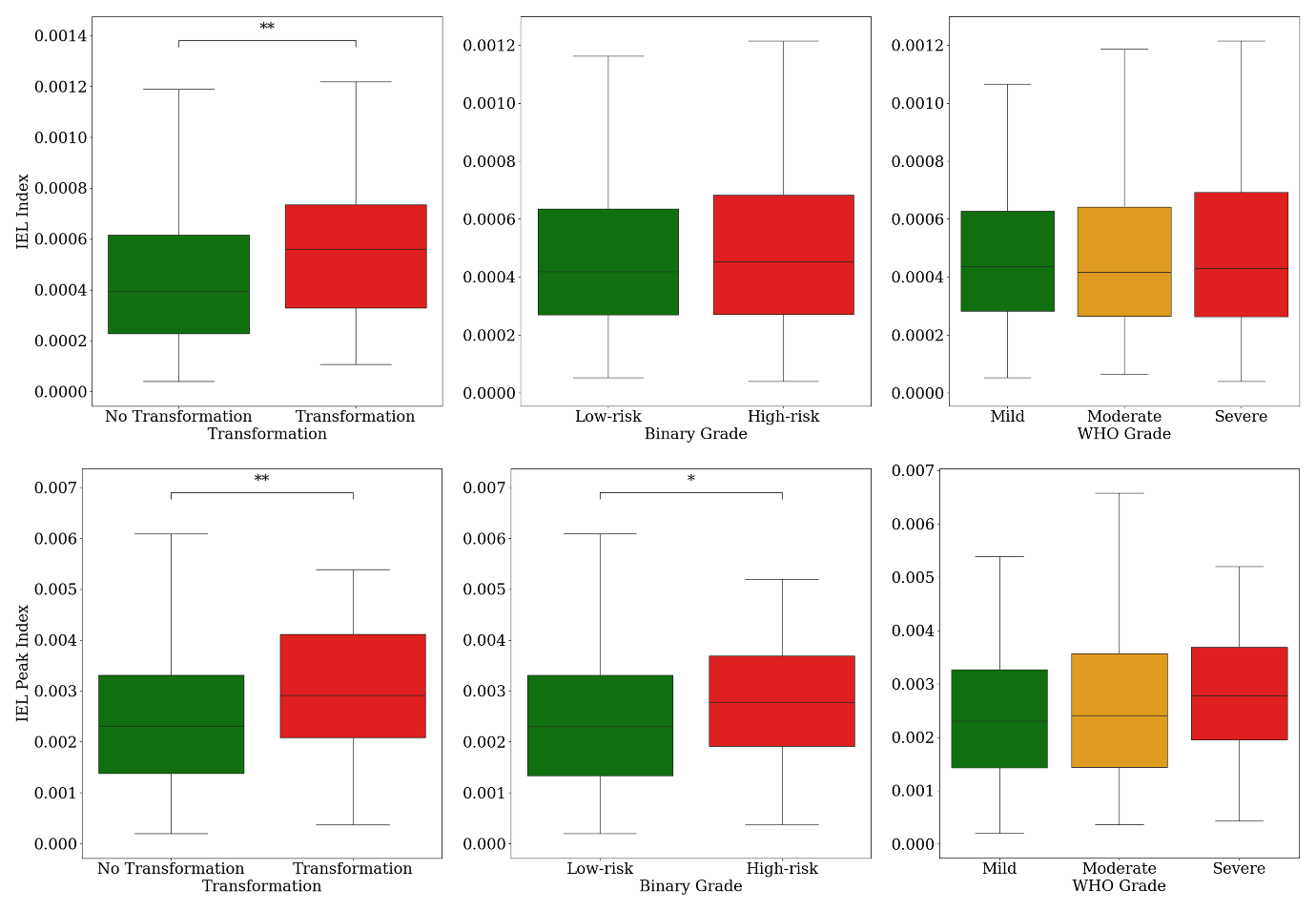


**Figure S2.** Boxplots showing the distribution of IEL scores in OED cases according to: transformation status (left), where transforming cases are red and not transforming are green; binary grade (middle), where low-risk cases are green and high-risk are red; and WHO grade (right), where mild cases are green, moderate orange, and severe are red. The top row is for the IEL-I score and the bottom row the IEL-PI score.


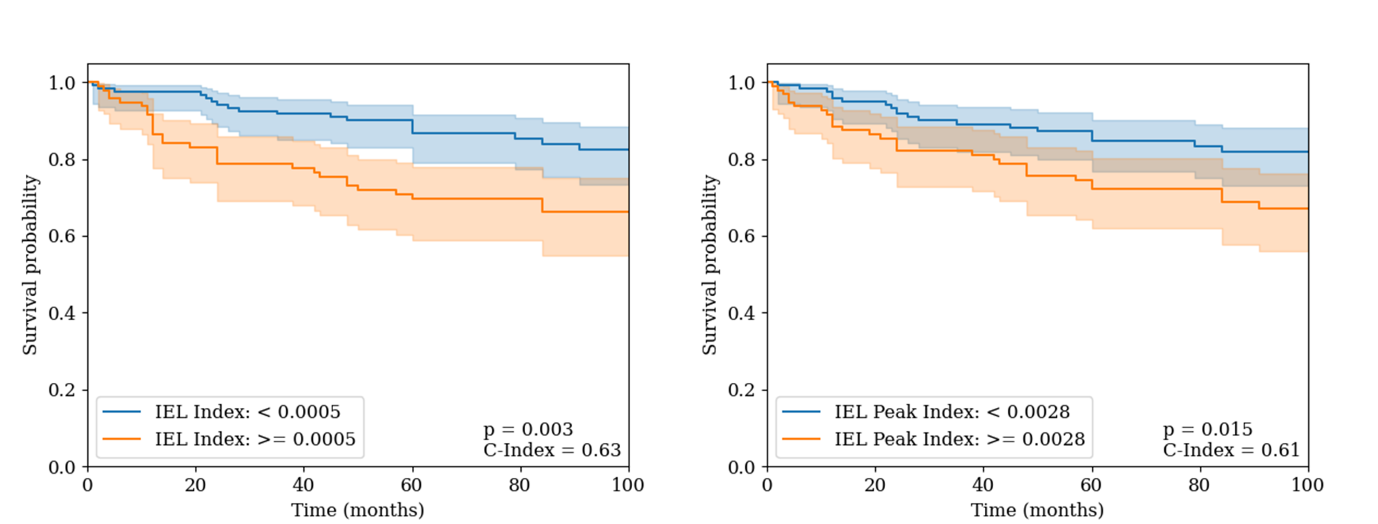


**Figure S3.** Kaplan Meier survival curves for IEL-I (left) and IEL-PI (right).

**Supplementary References**

1. Mahadeva S, Wyatt JI, Howdle PD. Is a raised intraepithelial lymphocyte count with normal duodenal villous architecture clinically relevant? J Clin Pathol. 2002;55(6):424–8.

2. Serra S, Jani PA. An approach to duodenal biopsies. J Clin Pathol. 2006;59(11):1133–50.
